# Motivations and deterrents of blood donation among blood donors during the COVID‐19 pandemic in Hong Kong

**DOI:** 10.1111/hex.13626

**Published:** 2022-10-17

**Authors:** Judy Yuen‐man Siu, Engle Angela Chan, Angus Siu‐cheong Li, Yik Mun Lee

**Affiliations:** ^1^ Department of Applied Social Sciences, Faculty of Health and Social Sciences The Hong Kong Polytechnic University Hong Kong People's Republic of China; ^2^ Interdisciplinary Centre for Qualitative Research The Hong Kong Polytechnic University Hong Kong People's Republic of China; ^3^ Research Centre for Sharp Vision The Hong Kong Polytechnic University Hong Kong People's Republic of China; ^4^ School of Nursing, Faculty of Health and Social Sciences The Hong Kong Polytechnic University People's Republic of China Hong Kong; ^5^ Hong Kong Red Cross Blood Transfusion Service Hospital Authority Hong Kong People's Republic of China

**Keywords:** barriers, blood donation, COVID‐19, current blood donors, Hong Kong, motivation, pandemic

## Abstract

**Background:**

The COVID‐19 pandemic has resulted in a reduction in blood donations and limited blood supply in many countries. The theory of planned behaviour has been widely used in past studies to understand the factors influencing blood donation. However, this theory limits analyses to the individual level. Furthermore, most research on the determinants of blood donation during the COVID‐19 pandemic is quantitative in nature, with relevant qualitative research being rare.

**Objectives:**

To investigate the motivators and demotivators for donating blood among current blood donors during COVID‐19 pandemic.

**Design:**

Forty in‐depth, individual semistructured interviews were conducted with current blood donors from December 2020 to March 2021 in Hong Kong. Thematic content analysis was adopted in the data analysis.

**Results:**

The majority of the participants (*n* = 37) were demotivated from donating blood during the COVID‐19 pandemic. Factors at the perceptual, social and institutional levels interacted to cause this reluctance. Only three participants felt more motivated to donate blood. The data revealed that sociocultural forces and government pandemic prevention policies strongly affected the participants' motivations to donate blood during the pandemic.

**Conclusion:**

This study presents a macro understanding of blood donation behaviour by investigating the institutional, social and perceptual factors influencing current blood donors during the COVID‐19 pandemic. This adds a more comprehensive understanding of blood donation where the theory of planned behaviour is widely used in past studies.

**Public Contribution:**

The participants shared their experiences in the interviews. Their experiences provide hints for explaining the decreasing blood donation during the pandemic times.

## 1 INTRODUCTION

COVID‐19 has posed substantial challenges to maintaining blood supply in numerous areas,^1^, ^2^, ^3^ including Hong Kong. This city has faced difficulty in collecting blood during the COVID‐19 pandemic.^4^ The blood supply has been depleted to a low level that is inadequate for the daily needs of hospitals^5^ as the pandemic got worse, from 300 to 400 units of donated blood collected daily since early January 2021,^6^ to 200 to 300 units collected daily since the beginning of March 2022 when the fifth wave of outbreak hit Hong Kong.^5^ The retention of current blood donors is one method of maintaining the blood supply. Haw et al.^1^ suggested that researchers must study how current blood donors have responded to the COVID‐19 pandemic and the motivations of their responses in the context of the pandemic, and how blood donors can be retained for continued donation in pandemic times.

In Europe, Africa, Brazil and China, participation in blood donation declined during the COVID‐19 pandemic.^7^, ^8^, ^9^, ^10^ Studies have found that the fear of contracting COVID‐19 is common among blood donors from numerous countries.^4^, ^7^, ^10^, ^11^, ^12^, ^13^ In Cameroon, donors were reluctant to donate blood if adequate safety measures were not in place at donor centres.^13^ In Saudi Arabia, people reported that they wanted to avoid contact with other blood donors to minimize their possibility of contracting the virus.^11^ Moreover, the adoption of the stay‐at‐home strategy was prominent for reduced blood donation in some African countries during the pandemic.^14^

### 1.1 Significance

The theory of planned behaviour has been widely used to understand the factors that influence blood donation.^15^, ^16^, ^17^, ^18^, ^19^, ^20^, ^21^ However, this theory is limited to the individual level of factors because it focuses on themes such as motives, self‐efficacy, anxiety and stress.^22^, ^23^, ^24^, ^25^, ^26^, ^27^ Blood donation behaviour should be understood as a multidimensional and nuanced construct grounded in a sociocultural context that influences decision‐making.^28^ Furthermore, most research on the reasons and challenges related to blood donation during the COVID‐19 pandemic is quantitative in nature, with relevant qualitative research being scarce. The few qualitative studies are mainly concerning the motivations and deterrents of blood donation in non‐COVID times in Uganda,^29^ Brazil,^30^ Malaysia^31^ and Britain.^32^ Altruism, helping others and having free check‐ups are noted as the key motivations,^17^, ^29^, ^30^, ^31^, ^32^ whereas fear of needles and blood, lack of awareness and access to blood donation facilities are noted as the key barriers.^17^, ^29^ Guglielmetti Mugion et al.^17^ further identify service quality and information and communication are key determinants for people to donate blood. Little is known about blood donation behaviours in Chinese communities during the COVID‐19 pandemic. Understanding how individual motivations to give blood are altered during a pandemic can assist health authorities in developing a socially and culturally responsive blood donation promotion strategy during future epidemics. Our research, thus, aimed to fill the aforementioned literature gap through an in‐depth investigation of the personal, social and institutional reasons for blood donors to donate and not to donate blood during the COVID‐19 pandemic.

## 2 METHODS

### 2.1 Study design

A qualitative approach involving 40 in‐depth, individual semistructured interviews was adopted to obtain data. Interviews were open‐ended to enable free‐flowing conversations between researchers and interviewees.^33^ The inductive nature of in‐depth interviews is effective for learning about people's beliefs, perceived meanings and interpretations.^34^

### 2.2 Data collection

Purposive sampling^33^ was conducted to recruit participants meeting the following criteria: (1) Hong Kong residents who lived and received education in Hong Kong, (2) aged 18–66 years, (3) had previously donated blood and intended to donate blood in the future and (4) did not donate blood during the COVID‐19 pandemic since January 2020. These selection criteria ensured the investigation of barriers to donating blood among blood donors during the COVID‐19 pandemic, and that the participants had extensive social exposure to the Hong Kong setting. The age range was set as 18–66 years because people of this age are eligible to donate blood in Hong Kong in accordance with the regulations of the Hong Kong Red Cross Blood Transfusion Service.

An interview question guide (see Supporting Information: Appendix 1) was used to maintain the focus of the interview while allowing an in‐depth discussion of topics.^34^ The topics of concern in the interview question guide, which were referenced from past literature about blood donation in epidemic times,^1^, ^4^, ^7^, ^8^, ^9^, ^11^, ^12^, ^13^, ^19^, ^35^ include interviewee perceptions, considering factors, motivations and barriers to donating blood during the COVID‐19 pandemic.

A total of 40 participants were recruited between December 2020 and March 2021. These participants were recruited in three phases. In the first phase, three individuals were selected from a survey pool at a local university. The survey pool was from an earlier cross‐sectional survey study on 542 undergraduate students about their knowledge and motivations in blood donation at a local university,^36^ of which 84 respondents had left contact details for the later interview study; 3 respondents were successfully contacted and agreed to be interviewed. Then in the second phase, recruitment posters listing the sampling criteria were placed at a local university's public facilities, such as the notice boards in the public areas of podium, activity areas and in canteens, with seven participants recruited. However, the use of these two strategies resulted in a sample of only university students and staff. Therefore, in the third phase, 30 participants with different demographic and socioeconomic backgrounds were recruited from the community, such as from social service centres, residents' associations and leisure and religious groups in different districts, in an attempt to minimize the selection bias.

To ensure interview consistency, each participant was individually interviewed by the same interviewer, who also served as a research associate of the study. The interviewer has bachelor's and master's degrees in sociology and qualitative research background. The interviewer received rigorous interview training from the first author before conducting the interviews. The first and second authors supervised and assisted the interviewer throughout the data collection process. The interviewer did not know the participants personally before to ensure the interviews were conducted with minimal bias. All the interviews were conducted in Cantonese, which is the native tongue of the interviewer and participants, to facilitate interactions. Probing questions were also asked to follow up on the participants' responses.

Each of the 40 interviews lasted 45–100 min. Because of the social distancing during the COVID‐19 pandemic, the majority of the interviews were performed online, and a few interviews were conducted face‐to‐face. The face‐to‐face interviews were conducted in a private room in the researchers' institution to ensure participant confidentiality. The interviews were audio recorded after obtaining the participants' consent. All the participants were given supermarket cash coupons with a value of 200 HKD (approximately US$26) upon their completion of the interviews to acknowledge their contribution.

### 2.3 Ethical approval

Ethical approval was obtained from the Human Subjects Ethics Subcommittee at Hong Kong Polytechnic University (reference number: HSEARS20171013002) before data collection. The study procedures were explained to each participant before their interview. The provided information sheets were prepared in traditional Cantonese (the participants' native language). Participants were assured that participation was voluntary. The participants in the online video interviews provided recorded verbal informed consent before the interviews, and the in‐person interviewees provided written informed consent. To protect participant privacy, no identifying information was collected in the audio or coded data. All the data were stored in password‐protected files, and interview transcripts were coded with anonymous identifiers to protect participant privacy. After the interviews had been transcribed, the audio recordings were destroyed.

### 2.4 Data analysis

Thematic content analysis was conducted to analyse the collected data. Interviews were transcribed verbatim. All interview transcripts were read and reread to achieve a general understanding and familiarity with the participants' experiences. Inductive coding was used to create coding schemes that allowed for the discovery of behaviour and thinking patterns.^33^, ^34^ A coding table with interview quotations was created to identify themes, categories and codes. After cleaning the interview transcript data, the transcriptions were divided into meaning units through abstraction and continual comparison. Codes were identified, and they were recorded in a coding table with supporting interview quotes. Similar codes were then compressed into categories and finally themes. New thematic codes that arose from the data as well as recurrent codes and themes were added to the coding table. Informant codes were added to the coding table to allow tabulation and the discovery of patterns. During the interview and coding process, memos were used to capture ideas and commentary. A codebook was used to record specific data.^33^ To guarantee the consistency and accuracy of the acquired data, the analytical procedures, coding and findings were documented in the codebook. To ensure that participant opinions were appropriately conveyed, direct interview excerpts were used, and the data analysis was grounded in these excerpts.

The first, second and third authors have extensive experience in qualitative research, and they conducted the coding separately; the first author is an anthropologist, the second author is a professor in nursing and registered nurse whose expertise lies in narrative research and the third author has a training background in sociology at both bachelor and master level. The qualitative research background of the first three authors ensured they could handle coding independently. Meetings were held among the first three authors every 2 weeks to discuss the coded data, and a consensus on the findings was reached. For publication, selected interview quotes were translated from Cantonese to English. Back‐translation was conducted to ensure that the meanings of the participant quotes were not distorted.

### 2.5 Participants

Table 1 presents the demographic information of the participants. All the participants (*N* = 40) had completed at least one blood donation before the pandemic (before January 2020) and planned to do so again in the future. All the participants had not donated blood since the COVID‐19 pandemic (from January 2020 onwards). The participants comprised 27 women and 13 men aged 23–65 years (*M* = 39 years) who had a wide range of educational and professional backgrounds. Thirty‐two participants had completed postsecondary, university or higher education, whereas eight had completed secondary school. The participants comprised 5 students, 8 retired or unemployed individuals and 27 employed individuals (either full‐time or part‐time). The total number of successful blood donations for each participant ranged from 1 to 128 at the time of this study.

**Table 1:** Participant characteristics

Informant code	Sex	Age	Educational level	Number of blood donations
CD001	F	41	University	10
CD002	M	40	Doctorate	7–8
CD003	F	39	Masters	9
CD004	F	23	University	4
CD005	F	24	University	9
CD006	M	23	University	1
CD007	F	23	University	11
CD008	F	25	University	>10
CD009	F	28	University	12–13
CD010	M	27	Masters	~20
CD011	F	23	Masters	3
CD012	M	25	University	3
CD013	F	34	University	3
CD014	M	64	Secondary school	128
CD015	F	53	Secondary school	>5
CD016	M	39	University	>10
CD017	F	35	Masters	1
CD018	M	24	University	~3
CD019	F	29	University	3
CD020	M	25	University	>8
CD021	F	42	Masters	25–30
CD022	F	26	Doctorate	4
CD023	F	56	University	5–10
CD024	M	57	Tertiary	3
CD025	F	27	University	~10
CD026	F	65	Secondary school	2
CD027	M	63	University	90
CD028	F	46	Secondary school	<10
CD029	M	56	Masters	1
CD030	F	42	Secondary school	<20
CD031	F	25	University	13
CD032	F	28	Masters	5
CD033	F	46	Tertiary	3–4
CD034	F	43	University	15–20
CD035	F	65	Secondary school	25
CD036	F	58	Secondary school	3–4
CD037	F	40	Masters	~30
CD038	F	52	Secondary school	6–8
CD039	M	57	University	>10
CD040	M	52	University	~30

*Note*: Tertiary education means postsecondary education other than university degree programmes.

Abbreviations: F, female; M, male.

## 3 RESULTS

Two themes of motivations and deterrents for donating blood during the COVID‐19 pandemic were identified from the interview data. A concept map shown in Supporting Information: Appendix 2 indicates the interacting relationship of the themes at perceptual, social and institutional levels to explain the deterrents of blood donation among the participants during the COVID‐19 pandemic.

### 3.1 Motivations to donate blood

Three participants reported a higher motivation to donate blood during the COVID‐19 pandemic. Factors involving perceptual, social and institutional levels explained their motivations.

#### 3.1.1 Perceptual level

##### Performing good deeds during the epidemic

Two participants perceived that donating blood helped them overcome a sense of helplessness and their negative experiences and feelings during the COVID‐19 pandemic, which provided them hope and more positive emotions.

I would like to donate blood more and take responsibility for the needs of society during this pandemic. The world is too chaotic, so the world really needs more people to do good deeds and provide hope to others at times like this. Blood donation is a good deed and it's something that everyone can do, so it's on my to‐do list. Since there's nothing else that I can do, why shouldn't I do something that I'm capable of? I'm just doing what I can. The world is getting worse and worse these days, and donating blood can help me overcome these bad feelings and make me happier. (CD001)

#### 3.1.2 Social level

##### Civic responsibility

For all these three participants, donating blood is a type of civic responsibility that is important during the pandemic time because they assumed that fewer people would donate blood during this time.

I thought that there would be even a greater need [for blood] because many people might be afraid of donating during the pandemic. However, accidents happen every day. The demand for blood won't stop because of COVID [COVID‐19]. So, I think that as a donor, it's my responsibility to donate blood in these hard times. I should offer my help if I can if many other donors are hesitant [to donate blood] during COVID. (CD031)

#### 3.1.3 Institutional level

##### Confidence in blood donation centres

Confidence and trust in blood donation centres was a key reason for these three participants to consider donating blood during the pandemic.

Although the COVID [COVID‐19] situation is serious these days, I believe that as long as you do your part, you're fine. If you still get infected, it's no one's fault. I trust the blood donation procedures. I really don't understand why I would be infected; the needle is new and there's no way for you to get infected. I believe that the staff there would clean stuff more thoroughly during COVID. They'd be even more careful and hygienic. I believe the staff is more concerned than we are. If there's a ‘blood donation virus cluster’, it'd be a nightmare for them. (CD026)

### 3.2 Deterrents to donate blood

Thirty‐seven participants reported that they were more demotivated to donate blood during the COVID‐19 pandemic. Factors at perceptual, social and institutional levels were interlocking to explain their demotivation.

#### 3.2.1 Perceptual level

##### Perceived higher risk in medical settings

Medical facilities and sites were thought to be ‘dangerous’ by 35 participants and their family members. The participants were hesitant to donate blood because blood donation centres were thought of as medical facilities.

Medical places seem dangerous because they might have more viruses or bacteria. That affects me a lot and especially affects my family. They're concerned that I want to go to these dangerous places just to donate blood. I am not particularly anxious but if my family is concerned, I should consider that. (CD027)

Although some participants recognized that blood donation centres are not hospitals, they believed that anything related to the medical system is ‘dangerous’.

Hospitals are dangerous places. I know donation centers aren't hospitals, but they're still medical facilities and there are nurses inside. Therefore, my impression is that they're related to hospitals, and so they're dangerous places and that it's better not to go there. (CD034)

Infection cases related to blood donors and donation centres reinforced the participants' perceptions that donation centres are ‘risky’.

I thought about going to donate blood a while ago. However, I didn't end up going because I heard that the staff or a donor got COVID [COVID‐19] from the news. Donor centers are a lot like clinics, so they're risky. Donors have to sit close together in the procedure, so you can never know if someone sitting next to you has COVID. (CD025)

##### Perceived higher risk of staff in donation centres

To the 35 participants, the staff in donation centres are part of the medical system and are thus perceived to be ‘risky’. This perception demotivated them from further blood donations.

I think that nurses are risky because they're often in touch with blood. You know, blood can transmit many diseases, so nurses are probably very risky people. I'm not sure if the nurse that helps me donate is infected. You know, she needs to sit very close to me. If she's infected, then I'll be in danger. (CD005)

##### Perceived risk of infection through blood and needle insertion

For 31 participants, donating blood was perceived as risky during the COVID‐19 pandemic because of the needle insertion involved in the invasive donation procedure. The resulting wound was perceived as a route for being infected.

Blood donation is invasive. And when I think of that, I think it's better not to do it now. It's dangerous. There's a hole [a puncture] after donation. Although they give you a bandage to cover it, still there's a small wound, and so there's a chance of infection. Viruses can enter your body through that hole, and you'll get infected. I'm worried about that, so I did not donate over these years of COVID [COVID‐19]. (CD033)

Another participant shared a similar perception. The donation puncture involves blood, which caused him to think about the common transmission routes of many other infectious diseases.

There's a great chance for you to get this virus via the respiratory tract or blood. Many infectious diseases are transmitted through blood. Also, at the Red Cross, they extract a drop of blood from your fingertip and cause a wound. When you touch things in public with the virus on them, you can easily get infected through a fingertip wound. So, I've been trying hard to avoid getting any wounds during COVID [COVID‐19]. (CD040)

#### 3.2.2 Social level

##### Sense of guilt if blood recipients are infected

To the participants, the fundamental goal of blood donation is helping others. However, blood donation during the COVID‐19 pandemic was perceived to violate this principle. Twenty‐seven participants were concerned about the potential negative effects of their donated blood on the recipients. The participants could not know whether they had been infected with COVID‐19 before giving blood, and they would feel guilty if they transmitted the virus to the recipients.

I don't know whether I'm infected, so I don't want to donate blood. I wouldn't donate because I'm not sure about my situation. I can never guarantee [that I'm not sick]. If I get infected and then give my blood to other people, then the virus could enter into someone else's body. That's not good. You know, people who need a blood transfusion are probably very sick, and if they get my blood and I'm infected without symptoms, then I'll make them even sicker. Blood donation should help people, but if it turns out to harm someone, then I couldn't forgive myself. (CD006)

This sense of guilt and worry about good intentions leading to bad outcomes was not uncommon among the participants. Such concerns could even affect some committed and enthusiastic donors. One participant who had made 128 successful blood donations reported that he had withdrawn from blood donation during the pandemic because he could never be sure whether he was infected with COVID‐19.

I am a taxi driver, and I meet lots of strangers every day; I can never know whether I've caught the virus or not. I don't want to harm other people with my donated blood. Therefore, my motivation [to give blood] has declined a lot since [the outbreak of] COVID [COVID‐19]. I can't feel sure that my blood is safe to give to other people. If you don't get tested for the virus before giving blood, and if you really are infected and someone gets your blood, then that patient would catch the virus from you and get infected. The patient may still die even the surgery is successful. In this case, you're not actually helping others; you're harming them. (CD014)

##### Collective responsibility for COVID‐19 infection

To all the 37 participants, avoiding infection with COVID‐19 is not an individual matter but a collective responsibility because of the Hong Kong government's infection control and quarantine policies. If the participants were infected, many others who have had contact with them or their blood would become involved, and the participants might be blamed.

If I donate blood after getting infected, then I would cause many people to be quarantined. First, the patient who received my blood would get infected. That patient would need to do mandatory virus testing many times and might need to be quarantined; those who contacted that patient, such as family members, doctors, nurses, other patients in the same ward, etc. would all need to do virus testing and may need to be quarantined too. Second, the staff in the blood donation center where I gave blood would be involved as well because they were in contact with me. They and their family members would also need to be quarantined and do mandatory virus testing. I would cause trouble for so many people. I don't want this to happen. Getting infected isn't just an individual matter; many others would be involved. To be more responsible to others, I think that not donating seems to be a better option, unless there is a method that can prove that the donors are virus‐free before donation. (CD035)

The collective responsibility nature of COVID‐19 infection could also involve their families if they get infected. As a result, the participants refrained themselves from exposing to any risk such as having blood donation to prevent their families from getting involved.

It's not only about yourself. It's not a big deal for me, but I don't want my family to bear the risk. Especially, my family members are old now, so I can't just think of myself without thinking about my family. I don't want them to worry, and don't want them to be quarantined and get infected. (CD034)

The collective responsibility of COVID‐19 could also arouse concerns from the participants' family members about their blood donation during the pandemic, serving as another demotivator for blood donation.

Many of my family members are older, so I don't want to give them any more pressure. I don't just mean that they disagree with me going to medical places, but it's also me personally. I think that I should protect them and be responsible for their health by avoiding high‐risk places. I don't want to expose myself to a known risk and make them get infected. (CD022)

##### Peer pressure

The negative response to blood donation during the pandemic from 12 participants' peer networks served as a strong demotivator for blood donation among them.

All my friends aren't motivated to donate. They all think that it's dangerous to donate now. They think that blood is dangerous and can make you infected. They also think that pneumonia [COVID‐19] is serious now, so it's better not to go. I think that their worries are reasonable, so I don't donate either. (CD010)

#### 3.2.3 Institutional level

Appeals from government health institutions to control infections influenced participants perceptions of donating blood during the COVID‐19 pandemic. Twenty‐two participants perceived blood donation as violating these appeals.

##### Perception of blood donation as involving potentially dangerous gathering

Blood donation during the COVID‐19 pandemic was viewed as ‘dangerous’ by the participants because they regarded donation as involving a gathering that not only potentially exposed them to infection but also violated the government's pandemic policies.

I think that [donation] involves a dangerous gathering because I… I'm not picking on donation centers, but the nature of blood donation inevitably attracts crowds, and that makes me worried. That's against the government's advice on avoiding crowds. I can ensure that I myself didn't go to any risky places beforehand, but I can't guarantee that other people would do the same. If people are gathering in the donation center, the risk of infection increases, so the donation center could be dangerous. You know, donors sit close to each other when they donate blood. (CD007)

Another participant also added the following:

The government and doctors always say that we should avoid crowds. Blood donation, however, involves a gathering, and it can be risky. They [staff at donation centers] don't want just one to two donors to show up. There might be other donors at the same time. Also, I need to lie on a bed that other donors have laid on before I did. If the earlier donor was infected, then I might get infected as well by lying on the same bed. Therefore, I think that [blood] donation isn't ideal during COVID [COVID‐19]. Blood donation can be dangerous these days, and it's not good to do because it seems to go against the advice from the government. If I get infected, it'd cause problems for me and my family, my friends, and my colleagues, and I might be blamed for not listening to the government. (CD013)

##### Blood donation as nonessential

In accordance with requests from government health institutions to stay at home, the participants avoided going out unless necessary. Twenty‐two participants perceived blood donation as unnecessary during the COVID‐19 pandemic.

I think that if the COVID [COVID‐19] situation remains this serious, or if the number of infections stays in the double digits every day, I won't go out unless it's necessary or to go to work. You can donate blood at any time when the pandemic becomes less serious or ends. It isn't urgent or essential right now. After all, the government is encouraging staying and working from home, so there's no point for me to go out for something that's not essential. Going out right now has the risk of getting infected. You might help others by donating blood; but if you get infected, then you'll need to be helped by others. That sounds silly to me. (CD013)

## 4 DISCUSSION

This study expands the existing literature on the study of blood donation behaviour in which the theory of planned behaviour has been the dominant approach of the literature.^15^, ^16^, ^17^, ^18^, ^19^, ^20^, ^21^ Instead of focusing on the individual level factors of attitude, subjective norms, behavioural intention, perceived behavioural control and perceived power noted by the theory^37^ that can explain blood donation behaviour,^22^, ^23^, ^24^, ^25^, ^26^, ^27^ our study suggests further that macro sociocultural forces and government's infection control appeals as institutional factors interacted with individual perceptual factors to cause the participants' reluctance of blood donation in the COVID‐19 pandemic.

Government infection control policies were critical in causing the participants to reduce blood donations. The finding of a study that people who respect appeals to reduce COVID‐19 infection are less likely to donate blood during the pandemic^7^ is in accordance with the relevant finding of our study. Government advice to stay at home and avoid crowds substantially affected the participants' motivation because of their belief that blood donation involves a dangerous gathering and is nonessential. Consistent with previous research,^11^ the crowdedness of blood donation centres and the fear of encountering potentially infected donors reduced the participants' willingness to donate blood. To relieve the donors' worries, blood donation agencies can provide a more spacious donation environment with fewer donors donating at the same time. Furthermore, based on the findings of higher motivation to donate by some participants because of their belief in an even higher demand for blood donors in the pandemic, blood donation agencies can therefore emphasize the essentiality of blood donation during pandemic times for the sake of effective operation of the medical system.

The medical system, including hospitals and medical personnel, was considered high risk by the participants. Although the participants were aware that blood donation centres are not hospitals, the centres were still perceived to be connected to the medical system and thus were perceived as ‘dangerous’ places during the pandemic. This perception resulted in reduced blood donation intention. The aforementioned stereotypes of hospitals have been observed in other cultures with similar demotivating effects on blood donation during the COVID‐19 pandemic.^12^ The participants also perceived blood as a contaminating agent because numerous infectious diseases are blood‐borne. This finding is in agreement with a similar finding obtained in another Chinese community.^10^ To increase motivation for blood donation during the pandemic, addressing the aforementioned perceptions by emphasizing the low risk of contracting COVID‐19 because of the blood donation procedure and the pandemic prevention measures being implemented in blood donation facilities may be beneficial.^19^, ^38^ Also, reassuring the nonblood‐borne nature of COVID‐19 transmission will be crucial for blood donors.

The collective responsibility to prevent COVID‐19 infection was also a crucial demotivator for the participants. They stated that contracting COVID‐19 not only affects them individually but can also cause others to be quarantined. Such collective responsibility has imposed pressure on the participants in their decision of blood donation. The decision of blood donation, thus, is no longer an individual will, but it has become a collective decision among the donors and their families. Collective responsibility for preventing the pandemic has been repeatedly stressed by the mass media, governments and public health communications.^39^, ^40^, ^41^, ^42^, ^43^ Donating blood without knowing whether one is infected was perceived to be potentially harmful to others, both in a physical sense and social sense (i.e., collective responsibility). Participants were concerned about feeling guilty if they passed on the infection through their donated blood. A study reported that emotions such as guilt and shame can be triggered in crises and can influence behaviour^44^; thus, blood donation agencies can consider offering rapid COVID‐19 tests to all donors before the blood donation procedure to overcome the aforementioned concerns.

Our data reveal that blood donation was sometimes used as a coping mechanism during the COVID‐19 pandemic. Although many studies suggest that people are less prone to donate blood during the COVID‐19 pandemic,^4^, ^7^, ^8^, ^9^, ^11^, ^12^, ^14^ our findings suggest that a few participants might be more prone to donate blood during this time. Echoed with a past study,^19^ trust and confidence in blood donation centres were also important to enhance donation motivation. Thus, emphasizing the precautionary measures adopted at blood donation centres to enhance people's trust and confidence may facilitate blood donor retention during the pandemic.

### 4.1 Limitations

The data were obtained from 40 current blood donors who did not donate blood during the COVID‐19 pandemic in Hong Kong. Additional research on those who donate during the pandemic may provide more information about donation motivations. The high percentage of the participants possessed at least a university degree also makes this study include mostly the ideas of those with a tertiary education level. Future research should consider interviewing those with a lower education level.

## 5 CONCLUSION

This study provides a macro understanding of blood donation behaviour among current blood donors in terms of institutional, social and perceptual factors influencing their donation motivations during the COVID‐19 pandemic in Hong Kong; thus, this study extends the relevant literature, in which individual and personal factors affecting blood donation have mainly been investigated using the theory of planned behaviour.

## AUTHOR CONTRIBUTIONS

Judy Yuen‐man Siu and Engle Angela Chan designed the study. Yik Mun Lee arranged the study logistics. Angus Siu‐cheong Li conducted the interviews. Judy Yuen‐man Siu, Engle Angela Chan and Angus Siu‐cheong Li analysed and interpreted the data, and were the contributors in writing the manuscript. All authors read and approved the final manuscript.

## CONFLICT OF INTEREST

The authors declare no conflict of interest.

## ETHICS STATEMENT

This study receives ethics approval from the Human Subjects Ethics Subcommittee at Hong Kong Polytechnic University (reference number: HSEARS20171013002).

## Supporting information

Supplementary information.

Supplementary information.

## Data Availability

The data of this project are not publicly available due to informants' confidentiality but can contact the corresponding author and be provided based on reasonable request.
