# Supplementary material for: Motivations and deterrents of blood donation among blood donors during the COVID‐19 pandemic in Hong Kong
Source: Health Expect. 2022 Oct 17;25(6):3192–201. doi: 10.1111/hex.13626 (PMC9700176; doi:10.1111/hex.13626)
Supplement: Supplementary file 1 — Supplementary information. [file HEX-25-3192-s002.docx]

**Appendix 1. Interview question guide**

1. What is the meaning of blood for you?
   1. How important do you think blood is?
2. What are the meanings of blood donation to you?
3. Did the meanings of blood and blood donation change since the COVID-19 pandemic?
4. What do you think about the act of donating blood?
   1. How important it is for one to donate blood?
   2. How important it is for one to donate blood in the COVID-19 pandemic?
5. Do any of your family members, friends, colleagues, classmates, etc. participate in blood donation?
   1. What do you think about their participation in blood donation? (probe: positive or negative experience)
   2. Does the participation of your family members, friends, colleagues, classmates make you more interested in or more likely to donate blood?
6. Why did you have your first blood donation?
7. Can you share your happy and unhappy experiences about blood donation?
   1. How do you think these experiences can motivate and/or demotivate you to donate blood?
8. Do you think your religious beliefs influence your thoughts about blood donation?
9. Can COVID-19 affect your thoughts in blood donation? If yes, how and in what ways?
10. Did COVID-19 affect your blood donation behavior, for example, did you donate more or less than ordinary times? Why?
11. Are you familiar with any promotion strategies to encourage blood donation (advertisement, etc.)? Can you talk about any that you are familiar with?
    1. Do any of these strategies make you feel more motivated to give blood? In what way?
12. What strategies do you think might work to increase blood donation in Hong Kong?
13. How do you feel about the blood donation centres and the blood donation process?
    1. Probe experience, environment, interaction with health care providers…
14. Have you ever engaged in other kinds of donation and/or charity behavior?
    1. If yes, can you compare these other kinds of donation and/or charity behavior with blood donation?
15. Do you have any other thoughts or ideas about blood donation?
