# Supplementary material for: Motivations and deterrents of blood donation among blood donors during the COVID‐19 pandemic in Hong Kong
Source: Health Expect. 2022 Oct 17;25(6):3192–201. doi: 10.1111/hex.13626 (PMC9700176; doi:10.1111/hex.13626)
Supplement: Supplementary file 2 — Supplementary information. [file HEX-25-3192-s001.docx]

Appendix 2. Concept map of the deterrents of blood donation during the COVID-19 pandemic

Perceived risky donation procedure

Perceived high risk of donation staff

Perceived high risk in medical setting

**Perceptual factors**

Peer pressure

**Social factors**

Sense of guilt if blood recipients get infected

Collective responsibility

**Institutional factor**

Government’s infection control appeals
